# Workload measurement for molecular genetics laboratory: A survey study

**DOI:** 10.1371/journal.pone.0206855

**Published:** 2018-11-27

**Authors:** Enrico Tagliafico, Isabella Bernardis, Marina Grasso, Maria Rosaria D’Apice, Cristina Lapucci, Annalisa Botta, Daniela Francesca Giachino, Maria Marinelli, Paola Primignani, Silvia Russo, Ilaria Sani, Manuela Seia, Sergio Fini, Paola Rimessi, Elena Tenedini, Anna Ravani, Maurizio Genuardi, Alessandra Ferlini, on behalf of the Molecular Genetics Working Group of the Italian Society of Human Genetics, SIGU

**Affiliations:** 1 Center for Genome Research, University of Modena and Reggio Emilia, Modena, Italy; 2 Department of Medical and Surgical Sciences, University of Modena ad Reggio Emilia, Modena, Italy; 3 Laboratory of Human Genetics, Galliera Hospital, Genoa, Italy; 4 Medical Genetics Laboratory, Tor Vergata Hospital, Rome, Italy; 5 Medical Genetics and Molecular Biology Unit, Synlab Italy, Brescia, Italy; 6 Dept. Biomedicine and Prevention, Medical Genetics Section, Tor Vergata University of Rome, Rome, Italy; 7 Medical Genetics, University of Torino, Dept. Clinical &Biological Sciences, Torino, Italy; 8 Medical Genetics, San Luigi University Hospital, Orbassano, Italy; 9 Genetics Laboratory Unit, Department of Obstetrics and Pediatrics, AUSL-IRCCS of Reggio Emilia, Reggio Emilia, Italy; 10 Department of Laboratory Medicine, Medical Genetics, Niguarda Ca' Granda Hospital, Milan, Italy; 11 Cytogenetics and Molecular Genetics Laboratory, Istituto Auxologico Italiano, I.R.C.C.S., Milan, Italy; 12 Genetica Medica—AOU "A. Meyer" di Firenze, Florence, Italy; 13 Medical Genetics Laboratory; Fondazione IRCCS Ca’ Granda Ospedale Maggiore Policlinico, Milan, Italy; 14 Unit of Medical Genetics Unit, Department of Medical Sciences, University of Ferrara, Ferrara, Italy; 15 Istituto di Medicina Genomica, Università Cattolica Del Sacro Cuore, Fondazione Policlinico Universitario A. Gemelli, Rome, Italy; CNR, ITALY

## Abstract

Genetic testing availability in the health care system is rapidly increasing, along with the diffusion of next-generation sequencing (NGS) into diagnostics. These issues make imperative the knowledge-drive optimization of testing in the clinical setting. Time estimations of wet laboratory procedure in Italian molecular laboratories offering genetic diagnosis were evaluated to provide data suitable to adjust efficiency and optimize health policies and costs. A survey was undertaken by the Italian Society of Human Genetics (SIGU). Forty-two laboratories participated. For most molecular techniques, the most time-consuming steps are those requiring an intensive manual intervention or in which the human bias can affect the global process time-performances. For NGS, for which the study surveyed also the interpretation time, the latter represented the step that requiring longer times. We report the first survey describing the hands-on times requested for different molecular diagnostics procedures, including NGS. The analysis of this survey suggests the need of some improvements to optimize some analytical processes, such as the implementation of laboratory information management systems to minimize manual procedures in pre-analytical steps which may affect accuracy that represents the major challenge to be faced in the future setting of molecular genetics laboratory.

## Introduction

New technologies in genomics are changing clinical practice, increasing the volume of genetic testing and, consequently, healthcare spending. Besides, the evolution of knowledge in genomics and in medical genetics has also elicited the need for accurate estimation of the cost impact of genetic testing on healthcare system in relation to clinical utility. In fact, with the rapid diffusion of next-generation DNA sequencing technologies into diagnostics, and the dramatic drop down of the cost of sequencing, genomics has started to pervade health care in the area of traditional genetic diseases as well as across the entire medical field, from preconception to aging.

Therefore, detailed knowledge of genetic testing costs, are mandatory to design efficient and optimized health policies in the near future.

Estimates of laboratory workload have been reported for some laboratory specialties, including microbiology [1], pathology and laboratory medicine [2]. Several documents on both clinical and laboratory workloads are present on the web (https://www.cap-acp.org/wkload.php).

In the medical genetics field, estimates have been undertaken for genetic counselling activities and cytogenetic diagnosis [3]. A time analysis of clinical workload linked to molecular genetic testing has been performed more than 20 years ago [4]. Sixteen Canadian genetic services were surveyed, and the following median times were calculated: 60 minutes for standard counselling, 15 minutes for follow up face to face consultation (case review), 10 minutes for a phone discussion, and 15 minutes each for letter, intermediate report and specimen set up. Molecular test interpretation was 10 minutes. Times were highly variable, depending on disease inheritance and on the number of clinical actions required.

More recently, Heald et al.[5] proposed that workload might be better articulated depending on the type of clinical service offered. They therefore calculated workload for general counselling, as well as for counselling related to cancer, cardiovascular, and prenatal genetic activities. They also concluded that non-clinical activities should be transferred to supporting staff (hospital technicians, nurses). Great variety in time measurements was observed for minimum total time spent, first or second counseling sessions, and general versus specific counselling.

Obviously, workload measurements are helpful if not necessary for predicting clinical resources, hospital staffing and personnel enrollment.

Molecular laboratory workload was briefly considered by Susan Steinhouse (https://ukgtn.nhs.uk/fileadmin/_migrated/tt_news/news_files/RCPath_article_MolUs.pdf), who described and commented on the UK National External Quality Assessment Service (NEQAS) for Molecular Genetics activities. Participating laboratories were asked to assign workload to the EQA cases. The output of this study showed significant differences across laboratories. “Workload units”, equating to one minute of laboratory work, were used for the measurements. This approach might be rather complex when many techniques (DNA extraction, PCR, sequencing, MLPA, genotyping, also counting the number of amplicons) are monitored.

The Italian Society of Human Genetics (SIGU) has a robust experience in collecting data and activities of the Italian Medical Genetic Services in order to describe the Medical Genetics national scenario. The last survey of medical genetic services in Italy was reported by Giardino et al. providing an overview of the activities over a 4-year period [6].

We surveyed the time workload related to the laboratory techniques routinely used for molecular testing including next generation sequencing (NGS). In particular, this is the first study providing this type of information for NGS procedures. The purpose was to provide time estimates of wet laboratory procedures in Italian molecular laboratories offering genetic diagnosis. Here we show the results, interpretation and considerations derived from this survey, highlighting the importance, specificity and peculiarity of diagnostic-oriented molecular laboratory activity and underlying how to improve genetic testing productivity, in line with the current requirements of public health.

## Materials and methods

A survey was launched by the Molecular Genetics Working Group via the Italian Society of Human Genetics (SIGU). The survey was addressed to all members affiliated with SIGU society. No specific criteria were applied for laboratory selection. Two different questionnaires were prepared: Survey A, containing 55 questions, was designed to gather information about workloads and hands-on times for standard basic molecular techniques, while survey B, including 41 questions, was focused on Next-Generation Sequencing (NGS). A total of 42 laboratories participated in survey A, while survey B was restricted to 18 laboratories performing NGS analyses.

Data collected included number and type of processed analyses with average hands-on times for different ranges of sample size workloads. Workload was examined for all molecular genetics procedures performed nationwide to diagnose genetic diseases. The following techniques were surveyed considering the hands-on-time for the manual procedures, even if requested by automated protocols: Nucleic Acid Extraction, PCR, Reverse dot blot, Multiplex Ligation-dependent Probe Amplification (MLPA), Sanger sequencing, NGS sequencing (gene panels and exomes). For these techniques hands-on-time for each step of the analysis protocol were considered and evaluated.

Data from responding laboratories were collected and analyzed using R statistical package (matrixStats, doBy, ggplot2). For each question, the analyses were performed only if responses were available from a minimum of two laboratories. Time-per-sample calculations were performed considering different ranges of sample size workflows, and an average sample size workflow was estimated. Extreme outlier time values (lower/upper quartile ±3*IQ; IQ: interquartile range) were discarded from statistical analysis.

This study did not require any ethical approval, since no human subjects or human biological material were used, and only the techniques and laboratory methods were analyzed. Consequently no informed consent from patients was taken within the study. All original material is supplied and can be downloaded as supplementary files: Survey modules: SURVEY A module English (S1 Survey), SURVEY A module original language (S2 Survey), SURVEY B module original language (S3 Survey) along with a translated copy of guidelines to complete survey (S1 File), and Survey raw data: SURVEY A Raw Data (S4 Survey), SURVEY B module and Raw Data English (S5 Survey)

## Results

The number of laboratories that returned questionnaires for Survey A and Survey B are 39 and 18, respectively. Among the laboratories that answered Survey A, 67% (n = 26) reported using NGS, 57.6% of which (n = 15) filled-out also Survey B. Three laboratories returned Survey B only. Overall, response was obtained from 42 different laboratories (24 Survey A, 15 Survey A and B, 3 Survey B). Considering the geographic distribution of responding laboratories, North and Central Italy are the most represented regions, with a north-to-south decreasing gradient already observed in previous studies [6]: 57.1% and 28.6% of laboratories are located in the Northern and Central regions, respectively, compared to 14.3% in the Southern regions. Of the 42 responding laboratories, 31% are affiliated with university hospitals, 31% with public (16.7%) or private (14.3%) research hospitals, 14.3% with public hospitals and 11.9% with universities. Private laboratories accounted for the remaining 7.1%.

**Fig 1:**
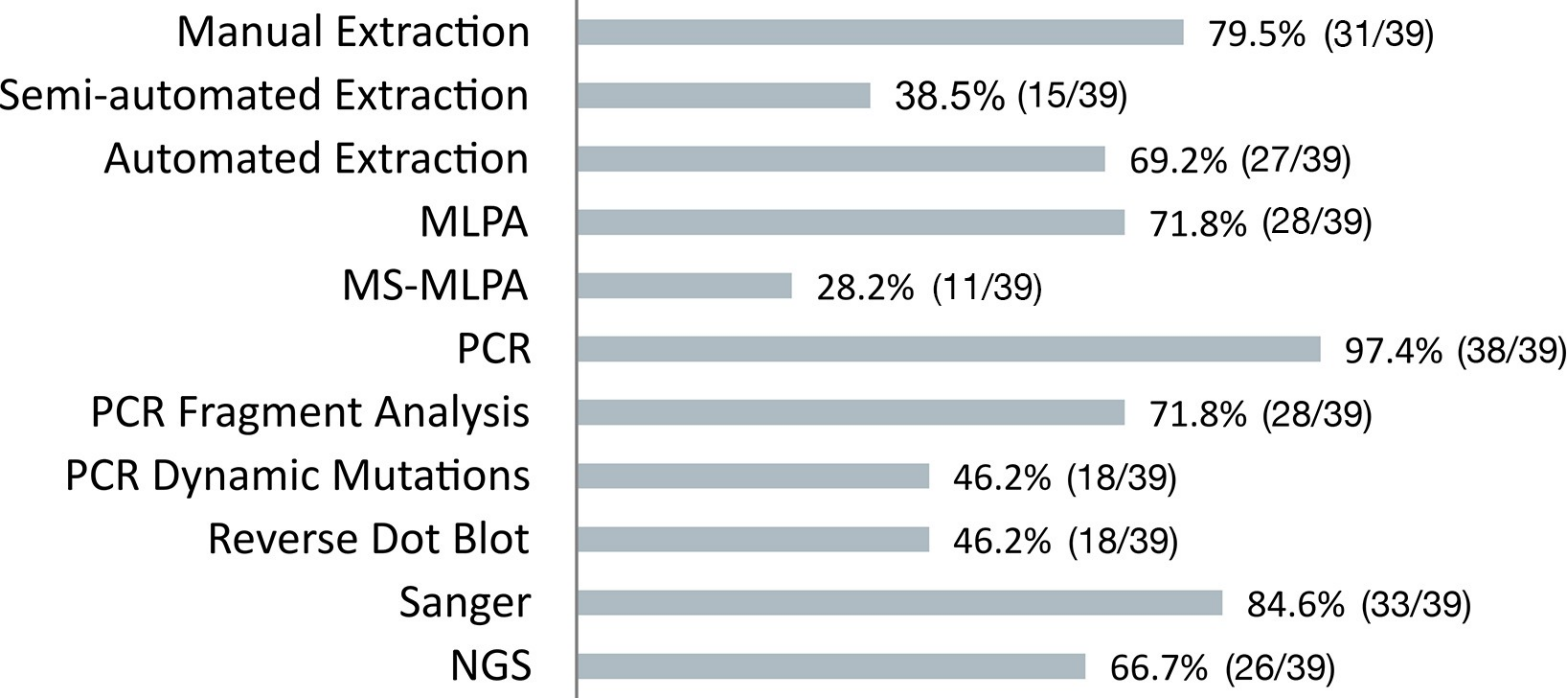
Fractions of labs performing each molecular procedure. Number (out of total) and percentage of labs performing each analytical procedure and who have completed the corresponding questionnaire sheets.

All responding laboratories reported information for each molecular activity. Fig 1 shows the fractions of labs performing each activity.

**Fig 2:**
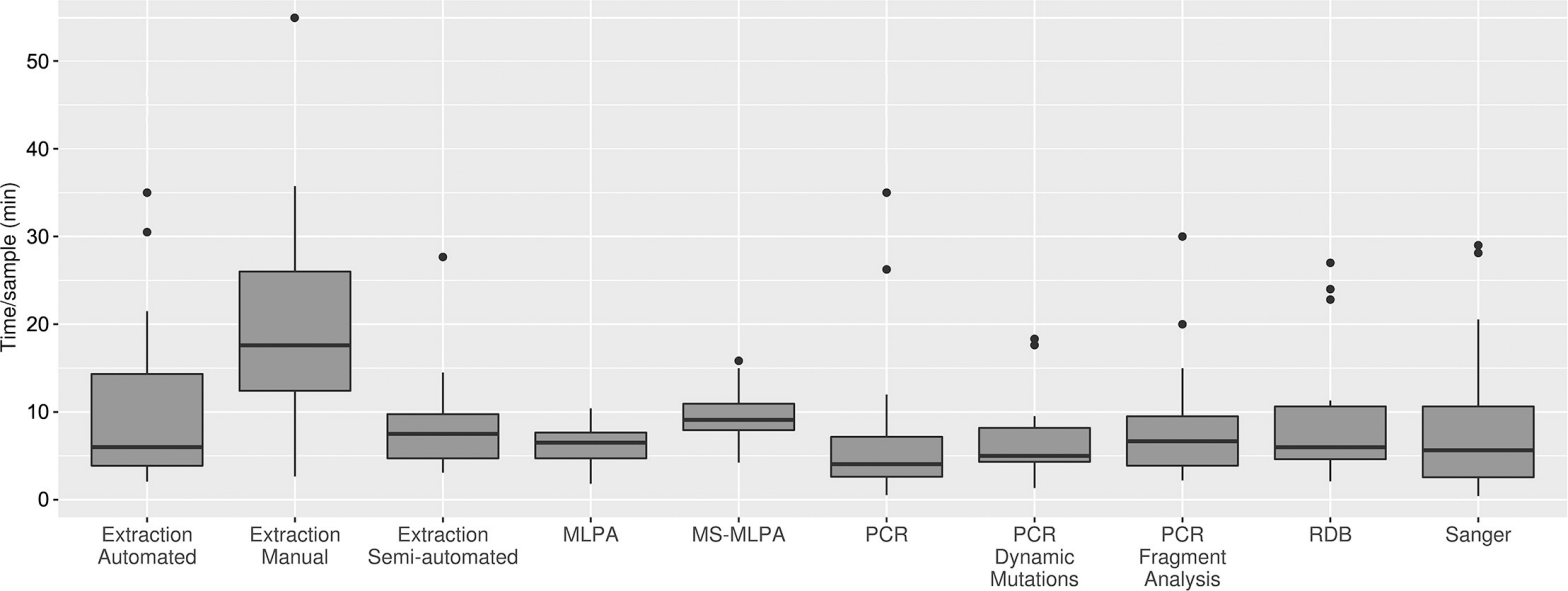
Hands-on times for each molecular procedure. Hands-on times for the molecular techniques surveyed. An average sample workload is considered. In box plots, center lines show the medians; box limits indicate the 25th and 75th percentiles as determined by R software; whiskers extend 1.5 times the interquartile range from the 25th and 75th percentiles, outliers are represented by dots. With regard to DNA extraction, "manual" refers to a basic technique that does not use kits and/or automation (e.g. Phenol-Chloroform); "semi-automated" refers to protocols in which commercial DNA extraction kits are used with simplified workflows, but no pipetting automation equipment is used; "automated" refers to a fully automated protocols implemented on liquid handlers platforms (e.g. Promega Maxwell, Qiagen QIASymphony etc.).

The summary of results of the data analysis of hands on times for the different molecular techniques considering an average workload are shown in Fig 2 and in Table 1. Sample DNA extraction analyses showed the greatest hands-on times, with also the greatest variation across laboratories for manual extraction (range 3–55 minutes/sample), suggesting, when compared with semi-automated and automated extraction (range 4–27 and 2–35 respectively) that these analyses are heavily operator- and method-dependent (differences in extraction methods have not been taken into account for hands-on times measurements).

**Table 1:** Hands-on times for each molecular procedure. Hands-on times for the different molecular techniques considering an average.

	Median (minutes)	Mean (minutes)	SD
Manual Extraction	17,85	19,88	11,2
Semi-automated Extraction	7,88	11,67	13,21
Automated Extraction	6	10,91	9,91
MLPA	6,4	6,5	2,3
MS-MLPA	9,1	9,48	3,45
PCR	4,05	5,97	6,51
PCR Fragment Analysis	6,67	7,85	5,99
PCR Dynamic Mutations	5	6,7	4,54
RDB	5,99	9,03	7,63
Sanger	5,7	9,66	11,16

Hands-on times for the molecular techniques surveyed. Median, Mean and Standard deviation are reported.

As expected, for all procedures hands-on times per sample decreased with increasing sample size (S1 Fig) [7].

**Fig 3:**
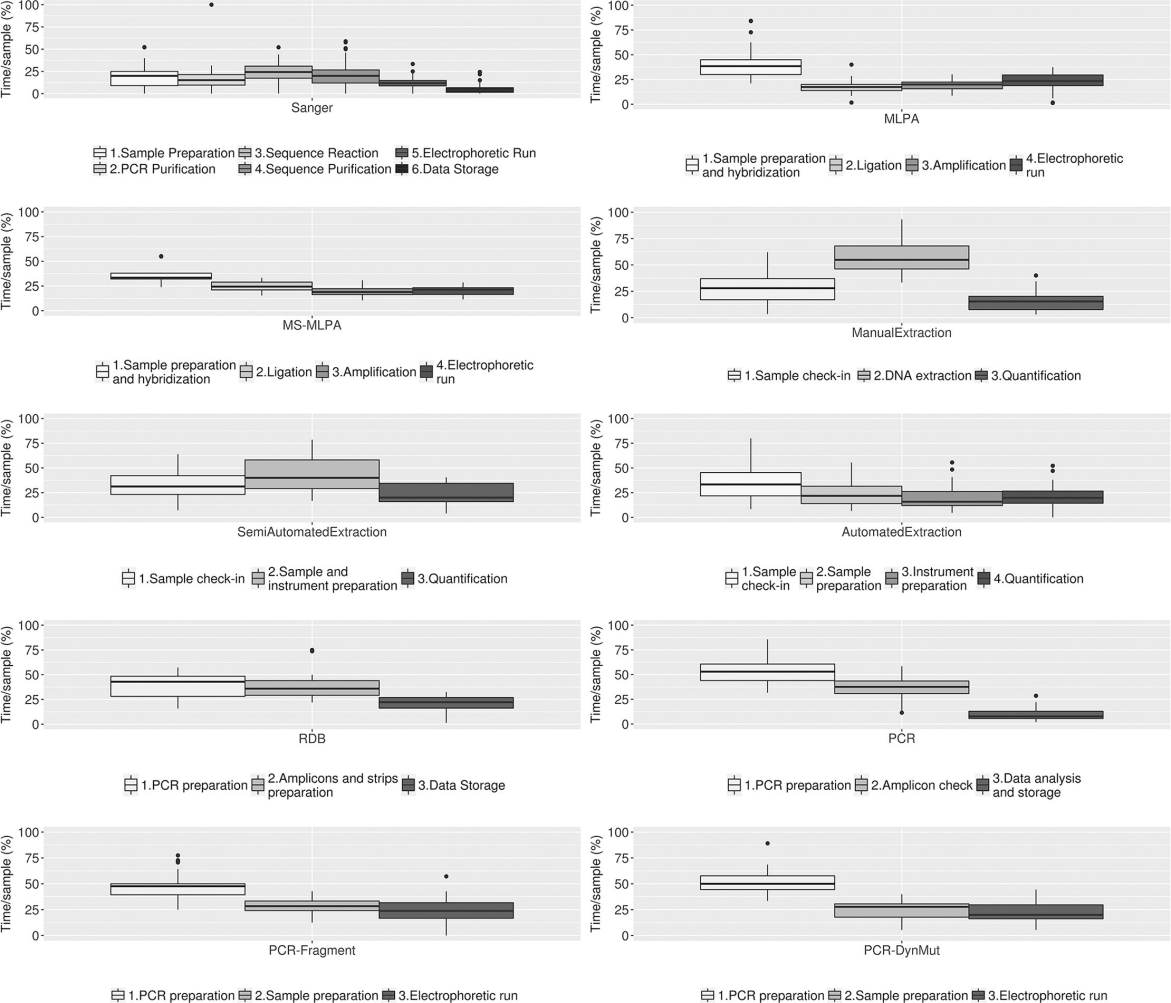
Hands-on times for each step of molecular procedure. Hands-on times for the different analytical steps of the molecular techniques surveyed. An average sample workload is considered. In box plots, center lines show the medians; box limits indicate the 25th and 75th percentiles as determined by R software; whiskers extend 1.5 times the interquartile range from the 25th and 75th percentiles, outliers are represented by dots.

Interestingly, when analytical steps were considered, for the large majority of molecular techniques, the most time-consuming steps are those that require an intensive manual intervention or in which the human bias can affect the yield in terms of time such as the pre-analytical phase, the preparation of daily worksheets and the reaction setup of the analytical session (Fig 3).

With regard to NGS, the responding laboratories reported workloads ranging between 20 and 300 samples/month (mean 90.05, median 55, SD 89.10). 66.6% of laboratories use an Illumina platform (Illumina Inc., San Diego, CA, USA (41%of them have both MiSeq and NextSeq/HiSeq) while 33.3% an Ion Torrent platform (Thermo Fisher Scientific Inc. Waltham, MA USA); 11% of laboratories use both Illumina and Ion Torrent platforms.

**Fig 4:**
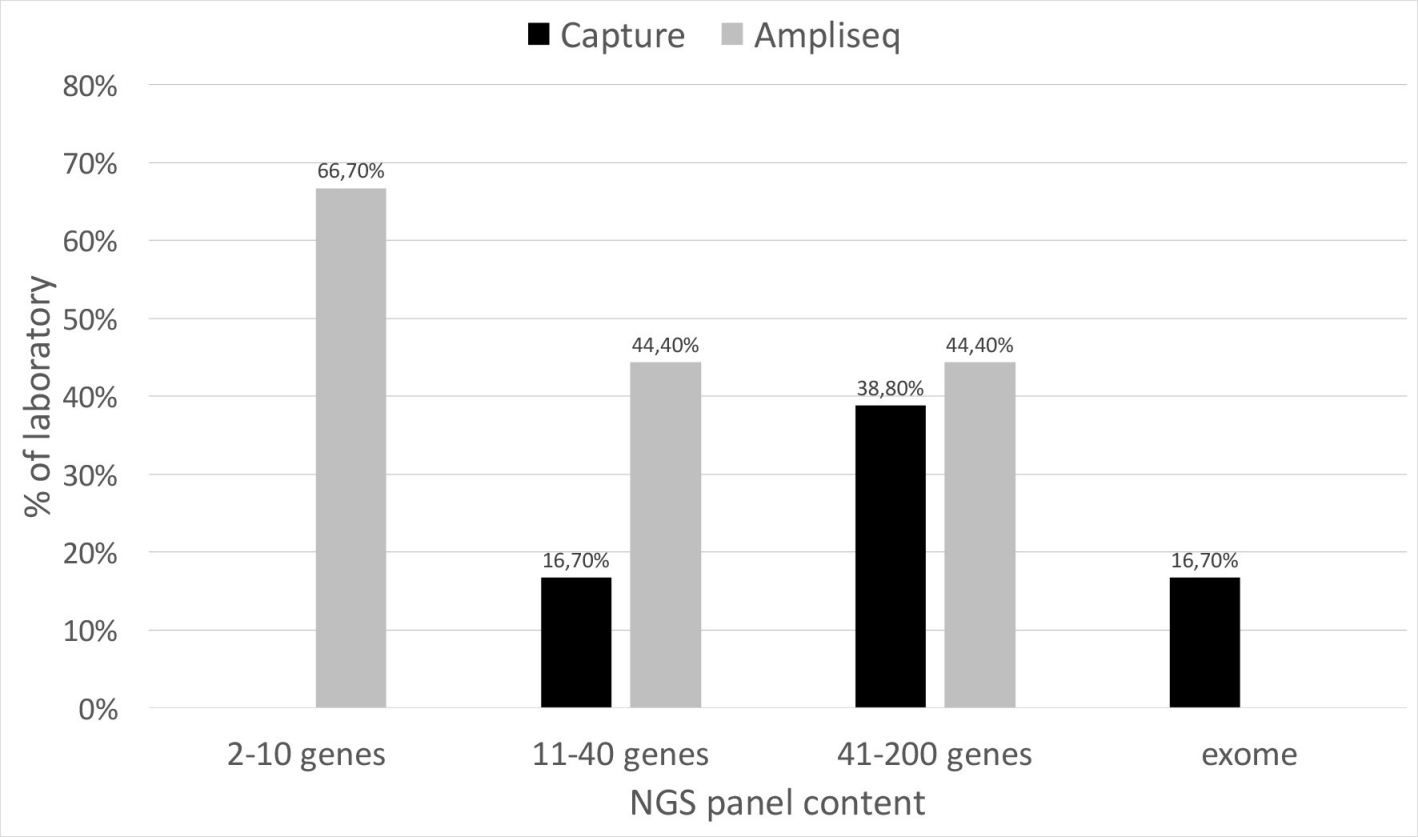
NGS strategies for target enrichment. Percentage of laboratory adopting the different strategies for target enrichment according to gene panel size.

Fig 4 shows the distribution of amplicon-based and capture-based strategies for target enrichment according to gene panel size. Overall, the amplicon-based technology is preferred to capture, mainly for small gene panels. Automated workflows were used by only one laboratory and 1/3 of the laboratories for capture-based target and amplicon-based target enrichment, respectively (data not shown).

**Fig 5:**
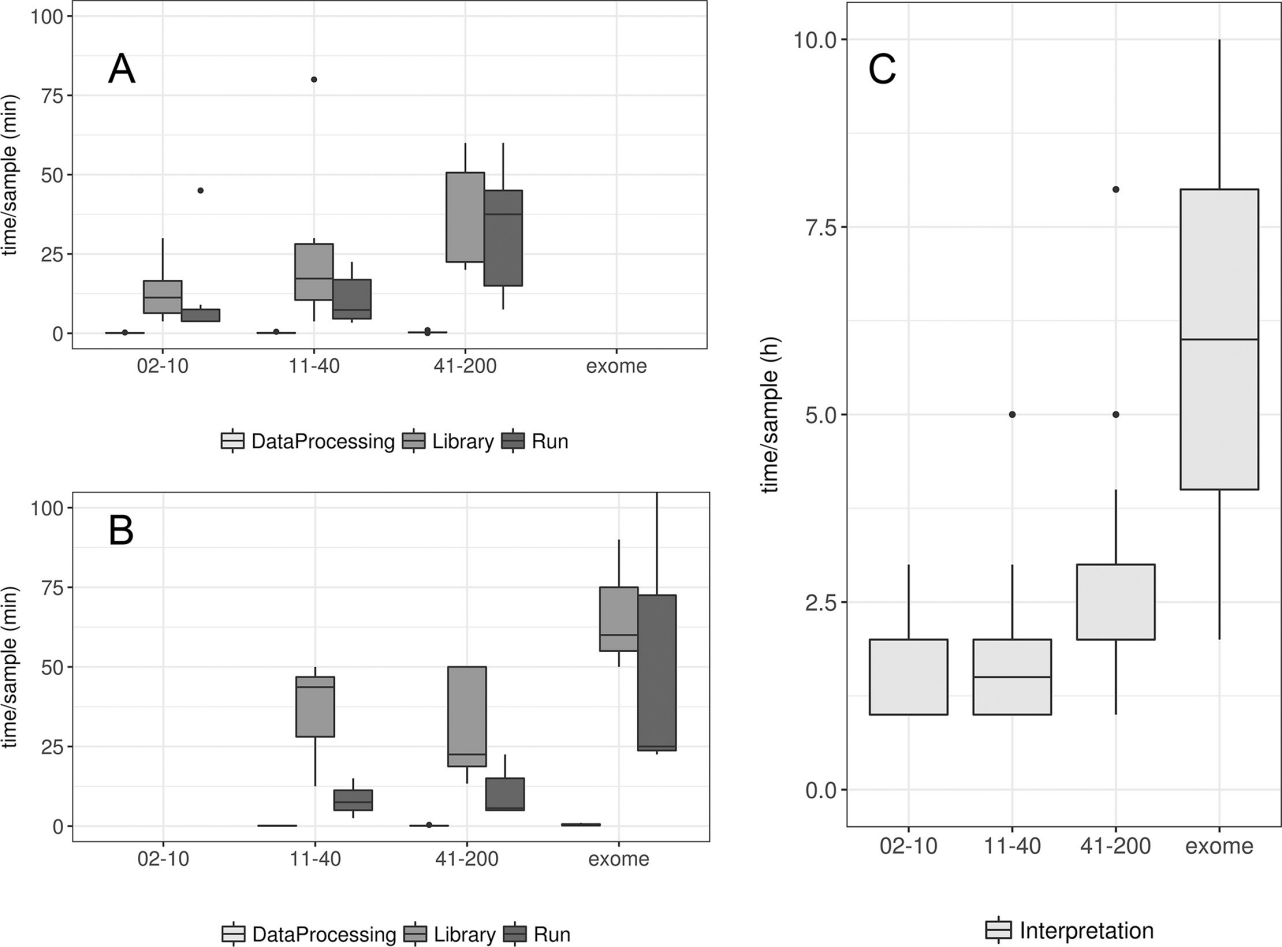
Hands-on times for NGS strategies. Hands-on times for NGS strategies. Panel A and B: amplicon- (A) and capture- (B) based technologies, considering library preparation, run set-up and raw data processing. Panel C: data interpretation. In box plots, center lines show the medians; box limits indicate the 25th and 75th percentiles as determined by R software; whiskers extend 1.5 times the interquartile range from the 25th and 75th percentiles, outliers are represented by dots.

The results of the data analysis of hands-on times for NGS are reported in Fig 5. Panel A and B show the hands-on times for amplicon- and capture-based technologies, respectively.

As expected, the most time-consuming step in the NGS analysis workflow is test interpretation. The time increases with the number of genes tested with an average of 1.6 hours requested for analyzing panels of 2–10 genes, 1.9 hours for 11–40 genes, 2.8 hours for 41–200 genes, and 6 hours for exomes (Fig 5C).

With regard to NGS data analysis pipelines, for small gene panels, the majority of laboratories use the software provided by the NGS platform, or in-house pipelines, whereas 30% use commercial software; for large panels and exomes, the implementation of in-house pipelines becomes predominant (80–100%). For data interpretation, in-house pipelines are the most used for intermediate gene panels (70–80%), whereas the use of commercial software increases with panel size (up to 50%), reflecting the need of additional tools for the interpretation of more complex and bigger amounts of data.

## Discussion

Molecular genetic diagnostic testing has become increasingly sophisticated during the last few years, mainly due to the breakthrough of genome and exome sequencing technologies. These play an increasingly important role in for highly penetrant diseases and cancer, allowing faster and more precise diagnosis, carrier testing of inherited disorders, family planning, and choice and monitoring of personalized therapies. Therefore, molecular genetic testing and clinical genomics represents one of the most powerful instruments in the personalized medicine era [8]. At the same time, molecular genetic laboratories must ensure high quality performances taking into account health-care provider requirements and patient management, numbers and types of biological samples, key challenges for each genetic/genomic technology, reporting turnaround times, optimization of working conditions for laboratory, and post-testing patient caring medical teams.

Management-engineering methods have been successfully applied to healthcare domains, such as clinical chemistry laboratories or clinical units/departments, using process design models to impact on those contexts. So far, these approaches have not been systematically applied to molecular genetics or clinical genomics laboratories. Therefore, there is a need to understand the peculiarities of the molecular genetics laboratories and the kind of tools and best practices that could be applied to improve this setting.

In order to fill in these gaps, we have undertaken the first survey describing the times requested for different molecular diagnostics procedures. The results obtained provide the grounds for an in-depth analysis of the operational workflows of molecular genetics laboratories.

Molecular genetics diagnostic testing is the translational output of research procedures and, even if it has become increasingly sophisticated, it has not yet reached the process optimization standards that are specific of high throughput laboratories, such as clinical chemistry laboratories. This survey allows some considerations that might be useful to optimize some analytical processes.

Our data strongly suggest that, for the large majority of molecular techniques, the highly time-demanding operations are those that require greater manual intervention or in which the human bias can affect the global process time-performances such as pre-analytical steps, and setup of the analytical sessions. For all laboratory procedures, the pre-analytical phase is currently considered as the weakest part of the testing process because of its effect on the global quality of the final results.

Moreover, the analysis of the times used in basic procedures (i.e. nucleic acid extraction and quality control along with sample acceptance and codification) suggests that their centralization, along with automation in shared work-units could help to implement efficiency and reduce personnel costs for these processes. In addition, these could be shared between laboratories working on nucleic acids, including microbiology/virology and pathology labs.

Laboratory automation is also relevant for its impact on the global quality of results, as it drastically reduces errors. In our study, we investigated the use of automated procedures for both standard molecular biology techniques and NGS. Automated DNA extraction was used by 69% of the laboratories. Conversely, in NGS procedures only one laboratory applied an automated workflow for capture-based target enrichment, just 1/3 of laboratories use automation for amplicon-based target enrichment and just 3 laboratories use automated liquid handling workstations for standard molecular biology procedures. We found that automated DNA extraction requires much shorter times, as expected. A similar tendency can be observed for standard molecular biology procedures and for NGS library preparation, although the number of responding laboratories is too low to draw definite conclusions. These data support again the need to centralize these procedures in order to optimize scale economy and operator related timing.

However, in order to globally improve laboratory automation and management, more efforts should be undertaken also by tech companies to adapt molecular genetics/genomics equipment to management engineering standards that are appropriate to other types of labs. Most of the instrumentation (especially those for NGS) cannot be interfaced with laboratory management systems (LIMS), thus still precluding the implementation of a complete automation process.

A result that deserves a possible further analysis comes from the percentages of use of each single molecular method. Virtually all laboratories perform, as expected, basic techniques. Interestingly, Sanger sequencing is performed by the 84.6% of responding laboratories (Fig 1) This shows on one hand that despite the fact that NGS is gaining ever-wider spaces in molecular diagnostics, Sanger sequencing still remains a key technology in molecular testing. Many are the reasons why this occurs. Indeed, some clinically well-defined conditions still require the targeted analysis (single gene diagnosis) of very limited portion of DNA sequence (i.e hemoglobinopathies/thalassemias or connexin-26-related hearing loss characterization) These are frequent Mendelian diseases due to mutations in small genes easily diagnosed by direct sequencing. In addition, Sanger is still appropriate for mutation screening in families where the genetic defect is known. Noteworthy, almost all guidelines for clinical NGS still require the Sanger validation of pathological variants.

Finally, prenatal diagnosis of Mendelian conditions is largely based on Sanger sequencing since causative mutations are already known but also when an urgent, not postponed, prenatal testing is needed (late pregnancy testing). As soon as noninvasive prenatal testing (NIPT) will be completely set up also for known Mendelian diseases, the usage of Sanger methods will be probably, at least for prenatal testing, reduced. It should be however underlined that the 15.6% of responding laboratories in our survey do not perform Sanger sequencing.

As far as the NGS is concerned, the survey shows that small gene panels are the main NGS approach for the investigation of genetic diseases. Recent analyses show an advantage in cost and in diagnostic power of whole genome sequencing (WGS) [9]and/or whole exome sequencing (WES) [10]compared to targeted sequencing using small gene panels. Despite the low number of responding laboratories, our results on WES analyses show that, as expected, working times are considerably higher compared to panel testing, mainly, though not only, due to longer times required for variant interpretation.

Indeed, our data show that, for all NGS strategies, the most time-consuming step in the NGS analysis workflow is variant interpretation. The costs of sequencing are decreasing very rapidly, faster than Moore's law (Wetterstrand K. DNA sequencing costs: data from the NHGRI large-scale genome sequencing program. http://www.genome.gov/sequencingcosts/). Conversely, manpower costs of downstream result analysis and interpretation requiring specialist knowledge is definitely increasing and represents a major challenge for the future of clinical genomics.

Although standards and guidelines for genetic test interpretation have been published in 2015[11], among the more than 7 million variants identified by the Exome Aggregation Consortium (ExAC)[12], less than 300,000 unique interpreted variants have been submitted to ClinVar by more than 630 laboratories, and one-third of these were classified by submitters as variants of uncertain significance. Some very interesting approaches have been proposed to support collaborative databases that systematically share genotype-phenotype correlations and variant interpretation data [13–18].

In this regard, the European Union funded the RD-Connect project (www.rd-connect.eu). This project is devoted to rare disease data collection and sharing including the NGS data in a unique large repository where data submission and storage may facilitate genome/exome comparison for diagnostic settings and test interpretation. Similarly, NIH in USA has launched a large collaborative action on rare diseases (RaDaR) with very similar purpose (https://ncats.nih.gov/radar)Finally, we found that there is wide variation in workloads (samples per month) for NGS analyses, with many laboratories processing low numbers of samples. This is in accordance with the very high number of genetic services and laboratories in some countries, including Italy, that also have an unequal distribution, with respect to the population size, as described for Italy by Giardino et al.(6) Although this might be understandable in view of the cultural and scientific background, some very rare diseases do need, it may have a negative impact on the optimization of analytical processes and costs. The European Union has recently approved the European Reference Networks (ERNs) for rare diseases (https://ec.europa.eu/health/ern_en). These ERNs now already established for 24 rare disease Networks and have the mission to harmonize the diagnosis and care of rare disorders, including genetic testing, across Europe. Wide discussion is ongoing within the ERNs about the possibility to optimize the molecular diagnosis of rare diseases in Reference centers that have a higher sample flow, together with excellent cultural knowledge about the rare disease(s). This is believed to optimize both analytical processes and their costs as well as a homogenous diagnostic offer for Rare Patients across Europe. The ERN participating centers (or Health Care Providers or HCPs), selected among various Excellence institutions in EU countries, are now entitled to design the best network profile in order to provide a comprehensive, equal and update standard of care and diagnosis, and new personalized therapies to all patients and families with rare genetic diseases. Nevertheless, this task is based on both Europe and Member States cooperation, but it also involves the entire Rare Disease community worldwide. Indeed, a stimulating challenge for the future of medical genetics.

In conclusion, we report as first a detailed workload calculation for the molecular genetics diagnosis activities via a survey in Italian laboratories. These estimations may serve to better evaluate the personnel effort need to optimize the molecular workflow, moving, when possible, toward high automation. These values may also have a significant impact in setting up personnel recruitment strategies by Hospitals. Costs represent a crucial issue to be faced in the Health care and are of outmost importance today, when the new NGS strategies have an increasing role and a consequent economic impact in the medical field.

## Supporting information

S1 Fig Hands-on times for standard PCR and the Sanger sequencing for increasing sample volumes. In box plots, center lines show the medians; box limits indicate the 25th and 75th percentiles as determined by R software; whiskers extend 1.5 times the interquartile range from the 25th and 75th percentiles, outliers are represented by dots.
(TIF)

S1 Survey A Module English. (DOCX)

S2 Survey A Module original. (DOCX)

S3 Survey A Original language. (XLSX)

S4 Survey A Raw Data. (DOCX)

S5 Survey A Module and raw data English. (XLSX)

S1 File Translated copy of guidelines to complete survey. (DOCX)
