# Supplementary figures and images for: Workload measurement for molecular genetics laboratory: A survey study
Source: PLoS One. 2018 Nov 27;13(11):e0206855. doi: 10.1371/journal.pone.0206855 (PMC6258511; doi:10.1371/journal.pone.0206855)

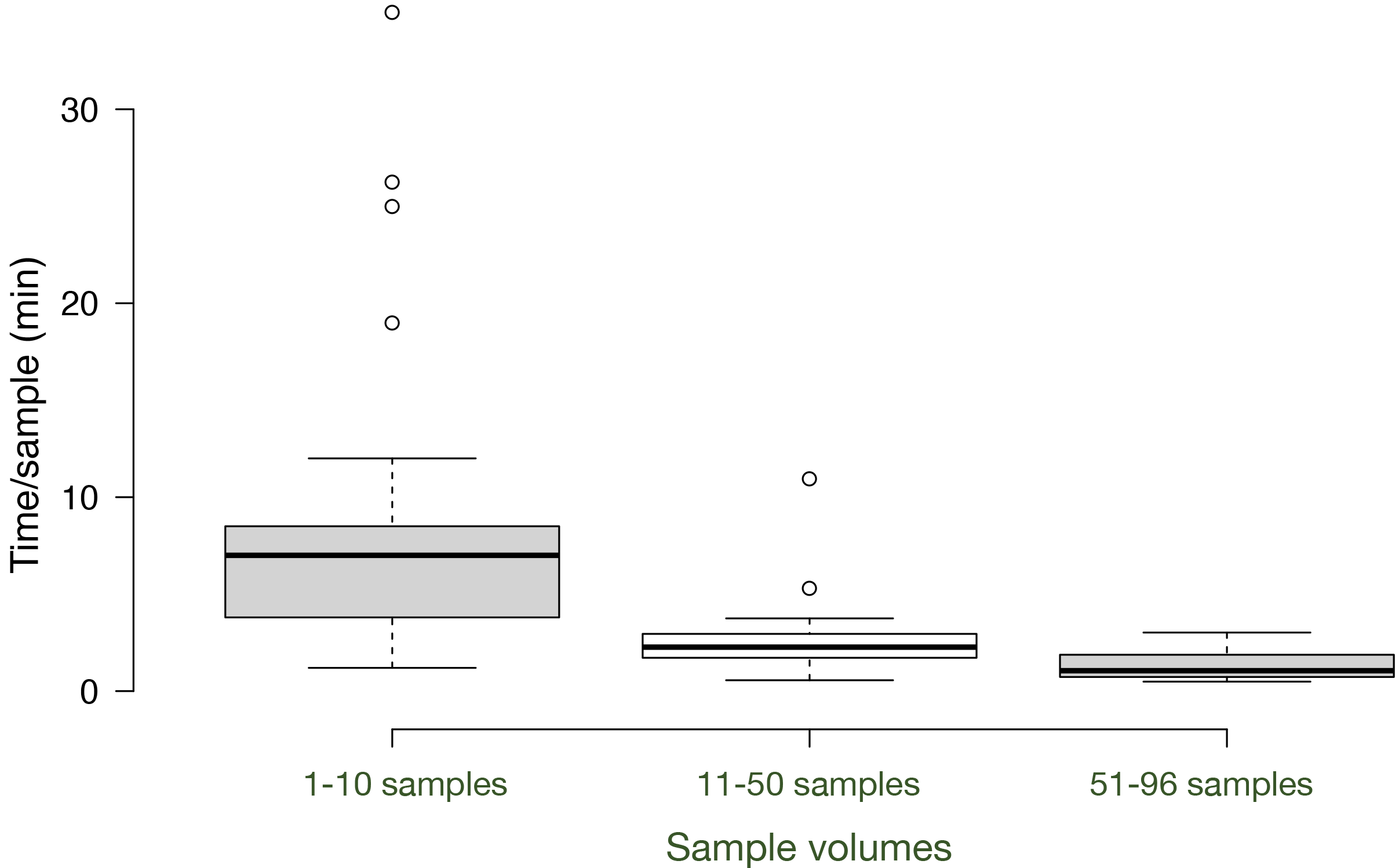

Supplement: S1 Fig — In box plots, center lines show the medians; box limits indicate the 25th and 75th percentiles as determined by R software; whiskers extend 1.5 times the interquartile range from the 25th and 75th percentiles, outliers are represented by dots. (TIF) [file pone.0206855.s001.tif]
