# Supplementary material for: Workload measurement for molecular genetics laboratory: A survey study
Source: PLoS One. 2018 Nov 27;13(11):e0206855. doi: 10.1371/journal.pone.0206855 (PMC6258511; doi:10.1371/journal.pone.0206855)
Supplement: S1 Survey A — (DOCX) [file pone.0206855.s002.docx]

**SIGU GDL MOLECULAR GENETICS**

**PROPOSAL OF SURVEY FOR ALL MOLECULAR GENETIC WORKERS MEMBERS**

**EVALUATION OF WORKLOADS IN MOLECULAR GENETIC LABORATORY**

**INTRODUCTION**

The Molecular GDL of SIGU considered important to produce a document concerning the calculation of the "wet" working times in the Laboratory of Molecular Genetics. Similarly to what is produced in Cytogenetics, the calculation of these times could be useful for the purposes of health planning and the calculation of the related fees.

For time calculation for NGS methods, "dry" times were also considered considering their relevance in the total time of the analysis.

Such a document does not exist at national level. The GDL used a small group that proposed the survey schemes and the parameters to be considered in the calculation of the times. The whole document was shared with the entire Molecular GDL, and then finalized and sent to the SIGU steering committee (CD) in the present form.

The questionnaire design was set up in a simple way to facilitate its maximum diffusion.

The times to be indicated are AVERAGE TIME, please do not enter intervals or ranges. In this case the average time will be calculated by the editors.

The Survey is not to be considered a census, but a module to have extensive participation and compilation in order to calculate hands-on-time for Italian molecular diagnostics laboratory in the most representative way

The data received can be processed to obtain average values.

**Manual DNA extraction:**

time verification scheme (Time expressed in minutes)

| **Procedure** | **Total time**  **(1 - samples)**  **(n: __) *** | **Total time**  **2-10 samples**  **(n: __) *** |  |
| --- | --- | --- | --- |
| Sample acceptance and coding | ......... ' | ..........' |  |
| DNA extraction | ......... ' | ..........' | Method:  ......... |
| Nucleic acid quantification | ......... ' | ..........' | Instrument:  ......... |

* indicate the exact number of samples

**Semiautomatic DNA extraction from peripheral blood:**

time verification scheme (expressed in minutes)

| **Procedure** | **Total time**  **(1 - 10 samples)**  **(n: __) *** | **Total time**  **11 - 20 samples**  **(n: __) *** | **OPTIONS** |
| --- | --- | --- | --- |
| Sample acceptance and coding | ......... ' | ..........' |  |
| Sample and instrument preparation and setup | ......... ' | ..........' | Instrument:  ......... |
| Nucleic acid quantification | ......... ' | ..........' | Instrument:  ......... |

* indicate the exact number of samples

**Automatic DNA extraction from peripheral blood:**

time verification scheme (expressed in minutes)

| **Procedure** | **Total time**  **(1 - 8 samples)**  **(n: __) *** | **Total time**  **12 - 24 samples**  **(n: __) *** | **Total time**  **25 - 96 samples**  **(n: __) *** | **OPTIONS** |
| --- | --- | --- | --- | --- |
| Sample acceptance and coding | ......... ' | ..........' | .......... ' |  |
| Sample preparation | ......... ' | ..........' | .......... ' |  |
| Instrument setup | ......... ' | ..........' | .......... ' | Instrument:  ......... |
| Nucleic acid quantification | ......... ' | ..........' | .......... ' | Instrument:  ......... |

* indicate the exact number of samples

**Standard PCR reaction:**

time verification scheme (expressed in minutes)

| **Procedure** | **Total time**  **(1 - 10 samples)**  **(n: __) *** | **Total time**  **11 - 50 samples**  **(n: __) *** | **Total time**  **51 - 96 samples**  **(n: __) *** | **OPTIONS** |
| --- | --- | --- | --- | --- |
| Work sheet preparation and PCR preparation  (calculations, reagent preparation, mix preparation and reactions or other) | ......... ' | ..........' | .......... ' | Automation  YES  NO  Instrument:  ......... |
| Display of PCR products  (eg: preparation and loading of agarose gel, EC or other) | ......... ' | ..........' | .......... ' | Automation  YES  NO  Instrument:  ......... |
| Detection and image acquisition | ......... ' | ..........' | .......... ' | Instrument:  ......... |

* indicate the exact number of samples

**PCR reaction and fragment analysis for dynamic mutations**

**(eg FRAXA - HD - FRDA - SCA):Total**

time verification scheme (expressed in minutes)

| **Procedure** | **Total time**  **(1 - 16 samples)**  **(n: __) *** | **Total time**  **17 - 32 samples**  **(n: __) *** | **OPTIONS** |
| --- | --- | --- | --- |
| Work sheet preparation and PCR preparation  (calculations, reagent preparation, mix preparation and reactions or other) | ......... ' | ..........' | Automation  YES  NO  Instrument:  ......... |
| Sample preparation for capillary electrophoretic run | ......... ' | ..........' | ......... ' |
| Capillary electrophoretic run set  -up (instrument set-up and loading times) | .........' | .......... ' | ......... ' |

* indicate the exact number of samples

**PCR reaction and fragment analysis for multiple loci multipli**

(UPD, Maternal cell contamination):

time verification scheme (expressed in minutes)

| **Procedure** | **Total time**  **(1 - 10 samples)**  **(n: __) *** | **Total time**  **11 - 50 samples**  **(n: __) *** | **Total time**  **51 - 96 samples**  **(n: __) *** | **OPTIONS** |
| --- | --- | --- | --- | --- |
| Work sheet preparation and PCR preparation  (calculations, reagent preparation, mix preparation and reactions or other) | ......... ' | ..........' | ..........' | Automation  YES  NO  Instrument:  ......... |
| Sample preparation for capillary electrophoretic run | ......... ' | ..........' | ..........' | ......... ' |
| Capillary electrophoretic run set  -up (instrument set-up and loading times) | .........' | .......... ' | .......... ' | ......... ' |

* indicate the exact number of samples

**Reverse Dot Blot:**

Wet time verification scheme (expressed in minutes)

| **Procedure** | **Total time**  **(1 - 10 samples)**  **(n: __) *** | **Total time**  **11 - 24 samples**  **(n: __) *** | **OPTIONS** |
| --- | --- | --- | --- |
| Work sheet preparation and PCR preparation  (calculations, reagent preparation, mix preparation and reactions or other) | ......... ' | ..........' | Automation  YES  NO  Instrument  ......... |
| setup (only in case of automation)  (preparation of solutions or other) | ......... ' | ..........' |  |
| Amplified setups and strips, and possible reagents  (in case of manual RDB addition and manual removal of individual reagents in different reaction steps, or other) | ......... ' | ..........' | Automation  YES  NO  Instrument  ......... |
| Storage of raw data  (striping, scanning or acquisition etc.) It is not to be considered as analysis of the data or its interpretation) | ......... ' | ..........' |  |

* indicate the exact number of samples

**Sanger Direct Sequencing:**

Wet Time Check Pattern (expressed in minutes)

| **Procedure** | **Total time**  **(1 - 10 samples)**  **(n: __) *** | **Total time**  **11 - 50 samples**  **(n: __) *** | **Total time**  **51 - 96 samples**  **(n: __) *** | **OPTIONS** |
| --- | --- | --- | --- | --- |
| Work sheet preparation and sample preparation  (calculations, reagent preparation, mix preparation and reactions or other) | ......... ' | ..........' | .......... ' | Automation  YES  NO  Instrument:  ......... |
| PCR purification | ......... ' | ..........' | .......... ' | Automation  YES  NO  Instrument:  .........  Plate purification  YES  NO |
| Sequence reaction  (calculations, reagent preparation, mix preparation and reactions or other) | ......... ' | ..........' | .......... ' | Automation  YES  NO  Instrument:  ......... |
| Sequence reaction purification | ......... ' | ..........' | .......... ' | Automation  YES  NO  Instrument:  .........  Plate purification  YES  NO |
| Capillary travel set-up  (instrument set-up and loading times) | ......... ' | ..........' | .......... ' |  |
| Raw data storage  (saving, file name) It is not intended as data analysis or its interpretation by SW) | ......... ' | ..........' | .......... ' |  |

* indicate the exact number of samples

**MLPA:**

time verification scheme (expressed in minutes)

| **Procedure** | **Total time**  **(1 - 8 samples)**  **(n: __) *** | **Total time**  **9 - 16 samples**  **(n: __) *** | **Total time**  **17 - 24 samples**  **(n: __) *** | **OPTIONS** |
| --- | --- | --- | --- | --- |
| Worksheet preparation and sample preparation, hybridization | ......... ' | ..........' | .......... ' | Automation  YES  NO  Instrument: |
| Ligation | ......... ' | ..........' | .......... ' | Automation  YES  NO  Instrument: |
| Amplification  (calculations, reagent preparation, mix preparation and reactions or other) | ......... ' | ..........' | .......... ' | Automation  YES  NO  Instrument: |
| Capillary electrophoretic run  setup (instrument set-up and loading times) | ......... ' | ..........' | .......... ' | Automation  YES  NO  Instrument: |

* indicate the exact number of samples

**MS-MLPA**

timing check (expressed in minutes)

| **Procedure** | **Total time**  **(1 - 8 samples)**  **(n: __) *** | **Total time**  **9 - 16 samples**  **(n: __) *** | **Total time**  **17 - 24 samples**  **(n: __) *** | **OPTIONS** |
| --- | --- | --- | --- | --- |
| Worksheet preparation and sample preparation, hybridization | ......... ' | ..........' | .......... ' | Automation  YES  NO  Instrument: |
| Ligation | ......... ' | ..........' | .......... ' | Automation  YES  NO  Instrument: |
| Amplification  (calculations, reagent preparation, mix preparation and reactions or other) | ......... ' | ..........' | .......... ' | Automation  YES  NO  Instrument: |
| Capillary electrophoretic run  setup (instrument set-up and loading times) | ......... ' | ..........' | .......... ' | Automation  YES  NO  Instrument: |

* indicate the exact number of samples
