# Supplementary material for: Workload measurement for molecular genetics laboratory: A survey study
Source: PLoS One. 2018 Nov 27;13(11):e0206855. doi: 10.1371/journal.pone.0206855 (PMC6258511; doi:10.1371/journal.pone.0206855)
Supplement: S2 Survey A — (DOCX) [file pone.0206855.s003.docx]

**SiGU GDL GENETICA MOLECOLARE**

**PROPOSTA DI SURVEY PER TUTTI I LAVORATORI GENETICA MOLECOLARE SOCI SIGU**

**VALUTAZIONE DEI CARICHI DI LAVORO NEL LABORATORIO DI GENETICA MOLECOLARE**

**PREMESSA**

Il GDL Molecolare SIGU ha ritenuto rilevante produrre un documento riguardante il calcolo dei tempi di lavoro “wet” nel Laboratorio di Genetica molecolare. Analogamente a quanto prodotto in Citogenetica, il calcolo di questi tempi potrebbe essere di utilità ai fini della programmazione sanitaria e del calcolo delle tariffe ad essi correlate.

Per il calcolo tempo uomo per metodiche NGS sono stati anche considerati tempi “dry” vista la loro rilevanza nel tempo totale della analisi.

Un documento del genere non esiste in ambito nazionale. IL GDL si è avvalso di un gruppo ristretto che ha proposto gli schemi del survey e i parametri da considerare nel calcolo dei tempi. L’intero documento è stato condiviso con l’intero GDL Molecolare, e poi finalizzato ed inviato al CD SIGU nella presente forma.

La compilazione è stata impostata in modo semplice per facilitarne la massima diffusione.

I tempi da indicare sono TEMPI MEDI, si prega di non inserire intervalli o range. In tale caso sara calcolato dai redattori il tempo medio.

Il Survey non è da considerarsi un censimento, ma un modulo per avere vasta partecipazione e compilazione al fine di calcolare i tempi uomo in modo massimamente rappresentativo della diagnostica molecolare italiana.

I dati ricevuti potranno essere elaborati per ottenere valori medi.

**Estrazione DNA manuale:**

Schema di verifica tempi (TEMPO espresso in minuti)

| **Procedura** | **Tempo Complessivo**  **1- campione**  **(n:__)*** | **Tempo Complessivo**  **2-10 campioni**  **(n:__)*** |  |
| --- | --- | --- | --- |
| Accettazione e codifica campione | ………’ | ……….’ |  |
| Estrazione DNA | ………’ | ……….’ | Metodo:  ……… |
| Quantificazione acido nucleico ottenuto | ………’ | ……….’ | Strumento:  ……… |

*indicare il numero di campioni effettivi

**Estrazione DNA semiautomatica da sangue periferico:**

Schema di verifica tempi (espressi in minuti)

| **Procedura** | **Tempo Complessivo**  **1-10 campioni**  **(n:__)*** | **Tempo Complessivo**  **11-20 campioni**  **(n:__)*** |  |
| --- | --- | --- | --- |
| Accettazione e codifica campione | ………’ | ……….’ |  |
| Allestimento campione e strumento | ………’ | ……….’ | Strumento:  ……… |
| Quantificazione acido nucleico ottenuto | ………’ | ……….’ | Strumento:  ……… |

*indicare il numero di campioni effettivi

**Estrazione DNA automatica da sangue periferico:**

Schema di verifica tempi (espressi in minuti)

| **Procedura** | **Tempo Complessivo**  **1-8 campioni**  **(n:__)*** | **Tempo Complessivo**  **12-24 campioni**  **(n:__)*** | **Tempo Complessivo**  **25-96 campioni**  **(n:__)*** | **OPZIONI** |
| --- | --- | --- | --- | --- |
| Accettazione e codifica campione | ………’ | ……….’ | ……….’ |  |
| Allestimento campione | ………’ | ……….’ | ……….’ |  |
| Allestimento strumento | ………’ | ……….’ | ……….’ | Strumento:  ……… |
| Quantificazione acido nucleico ottenuto | ………’ | ……….’ | ……….’ | Strumento:  ……… |

*indicare il numero di campioni effettivi

**Reazione di PCR standard:**

Schema di verifica tempi (espressi in minuti)

| **Procedura** | **Tempo Complessivo**  **1-10 campioni**  **(n:__)*** | **Tempo Complessivo**  **11-50 campioni**  **(n:__)*** | **Tempo Complessivo**  **51-96 campioni**  **(n:__)*** | **OPZIONI** |
| --- | --- | --- | --- | --- |
| Allestimento foglio di lavoro e preparazione PCR  (calcoli, preparazione reagenti, preparazione mix e reazioni o altro) | ………’ | ……….’ | ……….’ | Automazione  SI  NO  Strumento:  ……… |
| Visualizzazione prodotti PCR  (es: preparazione e caricamento gel di agarosio, CE o altro) | ………’ | ……….’ | ……….’ | Automazione  SI  NO  Strumento:  ……… |
| Rilevazione e acquisizione immagine | ………’ | ……….’ | ……….’ | Strumento:  ……… |

*indicare il numero di campioni effettivi

**Reazione di PCR e analisi frammenti per mutazioni dinamiche**

**(es. FRAXA – HD – FRDA - SCA):**

Schema di verifica tempi (espressi in minuti)

| **Procedura** | **Tempo Complessivo**  **1-16 campioni**  **(n:__)*** | **Tempo Complessivo**  **17-32 campioni**  **(n:__)*** | **OPZIONI** |
| --- | --- | --- | --- |
| Allestimento foglio di lavoro e preparazione PCR  (calcoli, preparazione reagenti, preparazione mix e reazioni o altro) | ……….’ | ……….’ | Automazione  SI  NO  Strumento:  ……… |
| Preparazione campione per corsa elettroforetica capillare | ……….’ | ……….’ |  |
| Allestimento corsa elettroforetica capillare  (tempi di allestimento strumento e caricamento) | ……….’ | ……….’ |  |

*indicare il numero di campioni effettivi comprensivi di eventuali relativi controlli

**Reazione di PCR e analisi frammenti per loci multipli**

**(UPD, esclusione contaminazione materna):**

Schema di verifica tempi (espressi in minuti)

| **Procedura** | **Tempo Complessivo**  **1-10 campioni**  **(n:__)*** | **Tempo Complessivo**  **11-50 campioni**  **(n:__)*** | **Tempo Complessivo**  **51-96 campioni**  **(n:__)*** | **OPZIONI** |
| --- | --- | --- | --- | --- |
| Allestimento foglio di lavoro e preparazione PCR  (calcoli, preparazione reagenti, preparazione mix e reazioni o altro) | ……….’ | ……….’ | ……….’ | Automazione  SI  NO  Strumento:  ……… |
| Preparazione campione per corsa elettroforetica capillare | ……….’ | ……….’ | ……….’ |  |
| Allestimento corsa elettroforetica capillare  (tempi di allestimento strumento e caricamento) | ……….’ | ……….’ | ……….’ |  |

*indicare il numero di campioni effettivi comprensivi di eventuali relativi controlli

**Reverse Dot Blot:**

Schema di verifica tempi (espressi in minuti)

| **Procedura** | **Tempo Complessivo**  **1-10 campioni**  **(n:__)*** | **Tempo Complessivo**  **11-24 campioni**  **(n:__)*** | **OPZIONI** |
| --- | --- | --- | --- |
| Allestimento foglio di lavoro e preparazione PCR  (calcoli, preparazione reagenti, preparazione mix e reazioni o altro) | ………’ | ……….’ | Automazione  SI  NO  Strumento:  ……… |
| Allestimento strumento (solo in caso di automazione)  (preparazione soluzioni o altro) | ………’ | ……….’ |  |
| Allestimento amplificati e strips, ed eventuali reagenti  (in caso di RDB manuale aggiunta e rimozione manuale dei singoli reagenti nei differenti step di reazione, o altro) | ………’ | ……….’ | Automazione  SI  NO  Strumento  ……… |
| Archiviazione dati grezzi  (attaccatura strisce, scansione o acquisizione o altro. Non è da intendersi come analisi del dato o sua interpretazione) | ………’ | ……….’ |  |

*indicare il numero di campioni effettivi

**Sequenziamento Diretto Sanger:**

Schema di verifica tempi (espressi in minuti)

| **Procedura** | **Tempo Complessivo**  **1-10 campioni**  **(n:__)*** | **Tempo Complessivo**  **11-50 campioni**  **(n:__)*** | **Tempo Complessivo**  **51-96 campioni**  **(n:__)*** | **OPZIONI** |
| --- | --- | --- | --- | --- |
| Allestimento foglio di lavoro e preparazione campione  (calcoli, preparazione reagenti, preparazione mix e reazioni o altro) | ………’ | ……….’ | ……….’ | Automazione  SI  NO  Strumento:  ……… |
| Purificazione della PCR | ………’ | ……….’ | ……….’ | Automazione  SI  NO  Strumento:  ………  Purificazione in piastra  SI  NO |
| Reazione di Sequenza  (calcoli, preparazione reagenti, preparazione mix e reazioni o altro) | ………’ | ……….’ | ……….’ | Automazione  SI  NO  Strumento:  ……… |
| Purificazione della reazione di sequenza | ………’ | ……….’ | ……….’ | Automazione  SI  NO  Strumento:  ………  Purificazione in piastra  SI  NO |
| Allestimento corsa capillare  (tempi di allestimento strumento e caricamento) | ………’ | ……….’ | ……….’ |  |
| Archiviazione dati grezzi  (salvataggio, nome file. Non è da intendersi come analisi del dato o sua interpretazione mediante SW) | ………’ | ……….’ | ……….’ |  |

*indicare il numero di campioni effettivi

**MLPA:**

Schema di verifica tempi (espressi in minuti)

| **Procedura** | **Tempo Complessivo**  **1-8 campioni**  **(n:__)*** | **Tempo Complessivo**  **9-16 campioni**  **(n:__)*** | **Tempo Complessivo**  **24-17 campioni**  **(n:__)*** |  |
| --- | --- | --- | --- | --- |
| Allestimento foglio di lavoro e preparazione campione, ibridazione | ………’ | ……….’ | ……….’ | Automazione  SI  NO  Strumento: |
| Ligazione | ………’ | ……….’ | ……….’ | Automazione  SI  NO  Strumento: |
| Amplificazione  (calcoli, preparazione reagenti, preparazione mix e reazioni o altro) | ………’ | ……….’ | ……….’ | Automazione  SI  NO  Strumento: |
| Allestimento corsa elettroforetica capillare  (tempi di allestimento strumento e caricamento) | ………’ | ……….’ | ……….’ | Automazione  SI  NO  Strumento: |

*indicare il numero di campioni effettivi comprensivi dei relativi controlli

**MS-MLPA**

Schema di verifica tempi (espressi in minuti)

| **Procedura** | **Tempo Complessivo**  **1-8 campioni**  **(n:__)*** | **Tempo Complessivo**  **9-16 campioni**  **(n:__)*** | **Tempo Complessivo**  **24-17 campioni**  **(n:__)*** |  |
| --- | --- | --- | --- | --- |
| Allestimento foglio di lavoro e preparazione campione, ibridazione | ………’ | ……….’ | ……….’ | Automazione  SI  NO  Strumento: |
| Ligazione | ………’ | ……….’ | ……….’ | Automazione  SI  NO  Strumento: |
| Amplificazione  (calcoli, preparazione reagenti, preparazione mix e reazioni o altro) | ………’ | ……….’ | ……….’ | Automazione  SI  NO  Strumento: |
| Allestimento corsa elettroforetica capillare  (tempi di allestimento strumento e caricamento) | ………’ | ……….’ | ……….’ | Automazione  SI  NO  Strumento: |

*indicare il numero di campioni effettivi comprensivi dei relativi controlli
