# Supplementary material for: Workload measurement for molecular genetics laboratory: A survey study
Source: PLoS One. 2018 Nov 27;13(11):e0206855. doi: 10.1371/journal.pone.0206855 (PMC6258511; doi:10.1371/journal.pone.0206855)
Supplement: S1 File — (DOCX) [file pone.0206855.s007.docx]

Dear SIGU Member

The Molecular GDL SIGU believes it is important to produce a document concerning the calculation of working time "wet" (operating at the counter) in the Laboratory of Molecular Genetics, similarly to what already produced in cytogenetics.

For the time calculation for NGS methods, "dry" times were also considered considering their relevance in the total time of the analysis.

The survey is not to be considered a census, but a module to have extensive participation and compilation in order to calculate hands-on-time for Italian molecular diagnostics laboratory in the most representative way.

The data received can be processed to obtain average values. The attached forms have been elaborated by the whole Molecular GDL and drafted and finalized by a drafting sub-group.

We sent the document we drafted to the SIGU steering committee, which approved it and accepted our proposal to share it with all the SIGU Members in order to collect as many comments as possible by filling out the questionnaire form.

A wide participation is fundamental in order to compose data reflecting reflect the activities iItalian laboratories.

We therefore ask you to fill in the attached as anonymous document following the instructions below:

- Fill in only the sheets of the **techniques actually used** in your laboratory or where you or your laboratory has direct experience
- Fill in the times considering **only the “lab-bench” activity**, thus excluding the time taken by the instruments or the time spent at the desk (to interpret the data or to prepare a report for example). To help you we have tried to describe in detail the activities considered "wet" and you find them specified under each step in brackets
- Only in the case of **NGS you are asked to also calculate the times for the "dry" activities** described in the specific questionnaire.
- Report only times **you can really measure**: time the real timing used in the procedure and then fill in the column with the number of real samples you were working on. For example: if you are timing your activity for the preparation of 20 samples fill in the 11-50 sample column and don’t fill also the 1-10 samples column calculating the timing based on what you do on the 20 samples
- Always indicate **how many samples you are processing** taking into account the control samples.
- Indicate the name of **instruments or automations** used (where required) so as to allow us to understand any significant variations in the estimate of the times
- The times to be indicated are **AVERAGE TIME**, please do not enter intervals or "range". In this case the average time will be calculated by the editors.

THANK YOU

Alessandra Ferlini
